# The Implications of an Ab Interno Versus Ab Externo Surgical Approach on Outflow Resistance of a Subconjunctival Drainage Device for Intraocular Pressure Control

**DOI:** 10.1167/tvst.8.3.58

**Published:** 2019-06-28

**Authors:** Richard M. H. Lee, Yann Bouremel, Ian Eames, Steve Brocchini, Peng Tee Khaw

**Affiliations:** 1 Chelsea and Westminster Hospital NHS Foundation Trust, London, UK; 2 National Institute for Health Research Biomedical Research Centre at Moorfields Eye Hospital NHS Foundation Trust and University College London (UCL) Institute of Ophthalmology, London, UK; 3 UCL Department of Mechanical Engineering, London, UK; 4 UCL School of Pharmacy, London, UK

**Keywords:** glaucoma, MIGS, ab interno, ab externo, surgery, GDD, IOP

## Abstract

**Purpose:**

Minimally invasive glaucoma surgery (MIGS) devices that drain into the subconjunctival space can be inserted via an ab externo or ab interno approach. Limited experimental data exists as to the impact of either technique on intraocular pressure (IOP) control. We performed microfluidic studies by using ex vivo rabbit eyes to assess the effect of each approach on outflow resistance of a subconjunctival drainage device for IOP control.

**Methods:**

A microfluidic experiment system was designed, consisting of a controlled reservoir of water connected to a pressure pump/flow sensor. The flow rate of water was fixed at 2 μl/min to simulate aqueous humor production. The pressure readings for each approach were recorded at a frequency of 1 Hz. A baseline reading was made before tube insertion into the eye (PEEK tube length set to aim for an initial outflow resistance of 5 to 10 mm Hg/μL/min) followed by measurements for a cumulative 2-ml volume entering the subconjunctival space. Results were adjusted for water viscosity at 37°C and reported as outflow resistance (mm Hg/μL/min ± standard error of mean).

**Results:**

Outflow resistance via the ab interno approach was 90.4% higher than with the ab externo approach being measured at 0.80 ± 0.11 mm Hg/μL/min and 0.42 ± 0.05 mm Hg/μL/min, respectively. Bleb formation was observed to be less predictable with the ab interno approach.

**Conclusions:**

The ab interno approach demonstrated greater outflow resistance and less predictable bleb formation than the ab externo approach. These results have implications for long-term IOP control and success depending on the approach to device insertion and could be an important consideration for future MIGS devices.

**Translational Relevance:**

The effect of the ab interno versus ab externo approach of a MIGS device inserted into the subconjunctival space was assessed. The ab interno approach demonstrated greater outflow resistance and less predictable bleb formation that may have implications for the development of future MIGS devices.

## Introduction

Glaucoma is the leading cause of irreversible visual loss worldwide and is associated with raised intraocular pressure (IOP).^1^,^2^ Surgical treatment options include glaucoma filtration surgery (GFS) or the insertion of a glaucoma drainage device (GDD). GDDs were traditionally used in eyes considered to be at high risk of failure following standard GFS (including neovascular glaucoma, uveitic glaucoma, and iridocorneal endothelial syndrome due to the increased risk of fibroblast proliferation and episcleral scarring).^3^ However increasingly positive results following GDD surgery have resulted in their increased use in lower risk patients as well. A recent Medicare study observed a 43% decrease in the number of trabeculectomies and a 184% increase in aqueous shunt surgery performed between 1995 and 2004 in the United States.^4^ However, despite the increased usage of GDDs, there are still complications associated with their use, including choroidal effusion, aqueous misdirection, suprachoroidal hemorrhage, decompression retinopathy, corneal edema, diplopia, and uncontrolled IOP requiring further intervention.^5^

Minimally invasive glaucoma surgery (MIGS), a term used to define a group of surgical procedures that involve a microincisional approach with minimal trauma to the target tissue, had a higher safety profile than conventional glaucoma drainage surgery and allowed for rapid recovery with minimal impact on the patient's quality of life.^6^ MIGS devices either modulate Schlemm's canal to improve trabecular outflow, facilitate the uveoscleral outflow by the development of a connection between the anterior chamber and the suprachoroidal space, or create an alternative outflow pathway into the subconjunctival space.^6^ The US Food and Drug Administration defined MIGS devices in their recent guidance as “A type of IOP lowering device used to lower IOP using an outflow mechanism with either an ab interno or ab externo approach, associated with little or no scleral dissection and minimal or no conjunctival manipulation.”^7^

The Xen gel stent (Aquesys Inc, Aliso Viejo, CA) is a 6-mm-long hydrophilic tube of porcine gelatin crosslinked with glutaraldehyde that is placed ab interno to optimize drainage into the subconjunctival space.^8^ Laminar flow through the device is calculated using the Hagen-Poiseuille equation, and its dimensions have been calculated to provide 6 to 8 mm Hg of flow resistance (with the Xen 45 implant) assuming an aqueous flow rate of 1.2 μL/min and normal aqueous humor viscosity, therefore reducing the risk of hypotony associated with conventional drainage devices.^8^ These findings have been corroborated with in vitro experimental flow studies that observed a steady-state pressure of 7.56 mm Hg at 2.5 μL/min vs. 0.09 and 0.01 mm Hg with the Ex-Press (Alcon, Fort Worth, TX) device and Baerveldt (BGI, Abbott Medical Optics, Santa Ana, CA) GDD, respectively.^9^

The InnFocus/PreserFlo MicroShunt (InnFocus Inc., Miami, FL) is also a tube (8.5-mm-long tube, 350 μm outer diameter and 70 μm inner diameter) that drains aqueous humor into the subconjunctival space but differs from the Xen gel stent as it is inserted via an ab externo approach and is composed of Poly (Styrene-block-IsoButylene-block-Styrene [“SIBS”]), a thermoplastic elastomer whose physical properties overlap both silicone rubbers and polyurethanes.^10^ Batlle et al.^11^ recently reported the 3-year results of a prospective nonrandomized study of 23 eyes with (*n* = 14) and without (*n* = 9) cataract surgery. Mean IOPs at 1, 2, and 3 years were 10.7 ± 2.8 (55%), 11.9 ± 3.7 (50%), and 10.7 ± 3.5 (55%) mm Hg, respectively. This is in comparison to the results of the Xen gel stent where studies have shown a mean IOP of 15 and 14.7 mm Hg at 1 year combined with or without phacoemulsification cataract surgery.^12^,^13^

According to the Hagen-Poiseuille equation, we would expect the pressure drop across the Xen gel stent and InnFocus Microshunt to be 10.3 and 2.5 mm Hg, respectively, at 2 μL/min. Given that these do not match the clinical outcomes reported, it was believed that factors other than the device dimensions may play a role in modulating the IOP control of these novel MIGS devices. The aim of this study was, therefore, to assess the role of device insertion (ab interno versus ab externo) on the outflow resistance of a subconjunctival drainage device. We performed microfluidic studies using ex vivo rabbit eyes to assess each approach and discuss the relevance of insertion technique in relation to the development of novel MIGS devices.

## Methods

### Tissue Preparation

Heads from freshly killed wild rabbits were obtained from a local butcher (F Conisbee & Son, Leatherhead, UK). Heads were shipped in a humid container and were approximately 36 hours from death to the start of the experiments. The eye and eyelid margin was exenterated to preserve the conjunctival layer as much as possible.

### Microfluidic Setup

**Figure 1:**
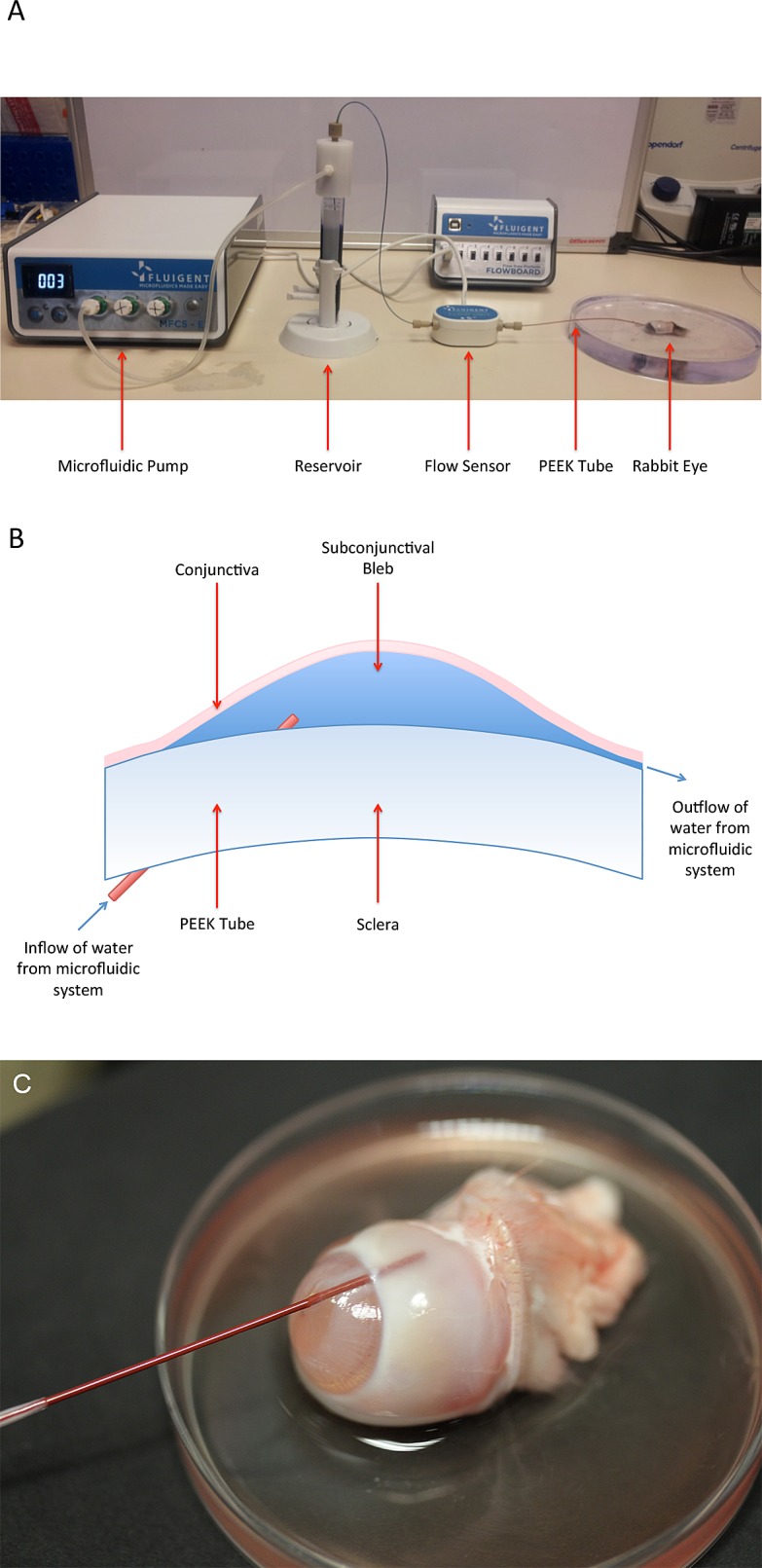
(A) Microfluidic setup demonstrating the pump pushing water from the reservoir through the flow sensor to the eye. (B) Schematic of fluid flow from the microfluidic setup into the subconjunctival space of the rabbit eye. (C) Close-up photograph demonstrating location of tube entering the eye through the cornea, into the anterior chamber angle and out into the subconjunctival space.

The ex vivo rabbit eye experiments were conducted at the University College London Institute of Ophthalmology with the experimental setup shown in Figure 1. A microfluidic approach was used, consisting of a reservoir of water connected to a pressure pump/flow sensor (Fluigent, Villejuif, France). The flow rate of water was fixed at 2 μL/min to simulate aqueous humor production, and the initial pressure to generate this flow rate was between 10 and 15 mm Hg to mimic normal IOP levels in human subjects. This was achieved by altering the length and diameter of the PEEK tube used in the microfluidic setup (Fig. 1A). Measurements of the outflow resistance before insertion into the eye were taken and this formed the baseline measurement for each eye. Outflow resistance via each surgical approach was recorded as the increase in pressure per μL/min (mm Hg/μL/min).

### Surgical Approach

The ab externo approach (*n* = 12) was performed as per conventional GFS/GDD surgery. A superior limbal conjunctival incision was created with Vanna scissors. Blunt dissection with Westcott scissors was performed to the level of the sclera and extended laterally and posteriorly. An 18G (green) cannula was used to create a channel through the center of the cornea, into the anterior chamber and exiting through the drainage angle into the subconjunctival space 3 mm posterior to the limbus. The cannula needle was exchanged for a 1/32-in outer diameter PEEK tube (internal diameter, 125 μm). The cannula was also removed, leaving the PEEK tube in situ running through the cornea and anterior chamber into the subconjunctival space, with the sclera forming a surrounding tight seal around the tube (Fig. 1C). This approach was assessed using blue dye to observe for any wound leak, and we did not observe any leakage of fluid into the anterior chamber or via the conjunctival incision made with the ab externo approach. Conjunctival closure was performed with 10/0 nylon (Ethilon, Ethicon, Somerville, NJ). The ab interno approach (*n* = 12) was performed in a similar fashion to the ab externo approach but without dissection and subsequent closure of the conjunctiva.

### Data Analysis

The experiments on each eye were conducted for 6 hours, and the pressure was recorded at a frequency of 1 Hz. A digital thermometer (Thor Labs, Newton, NJ) was used to record the temperature of the experiment with the same frequency of the microfluidic equipment, and results were adjusted for water viscosity at 37°C and reported as outflow resistance from within the subconjunctival space (mm Hg/μL/min ± standard error of mean). The difference in outflow resistance between the ab interno and ab externo approach was evaluated using an unpaired two-tailed *t*-test, and the level of significance was taken as *P* < 0.05.

## Results

**Figure 2:**
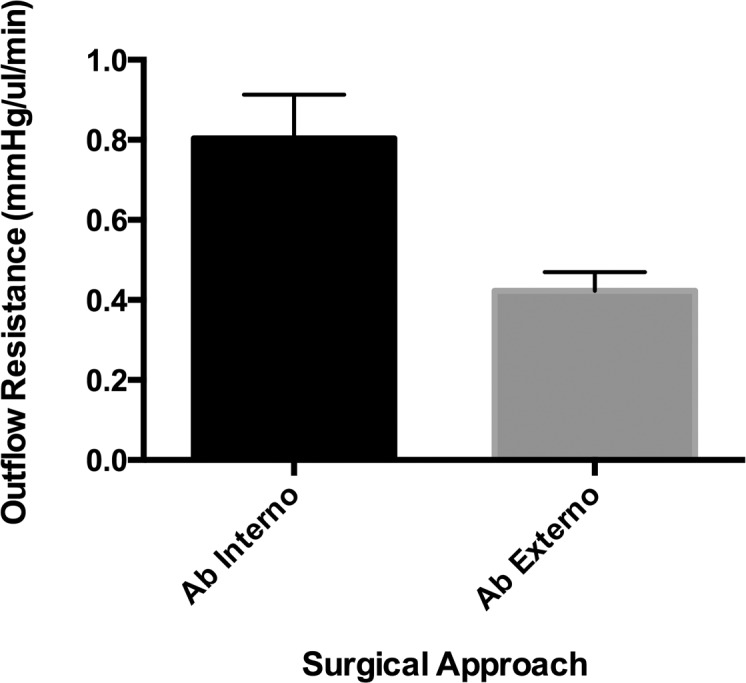
Graph of outflow resistance adjusted for water viscosity at 37°C via the ab interno and ab externo surgical approaches.

**Figure 3:**
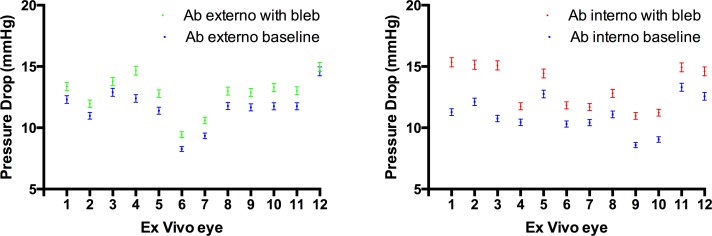
Difference in pressure drop between baseline and after bleb formation in each eye when the device was inserted via an ab externo (left) and ab interno approach (right). It is observed that the pressure drop via an ab interno approach is greater and more variable than via the ab externo approach.

**Figure 4:**
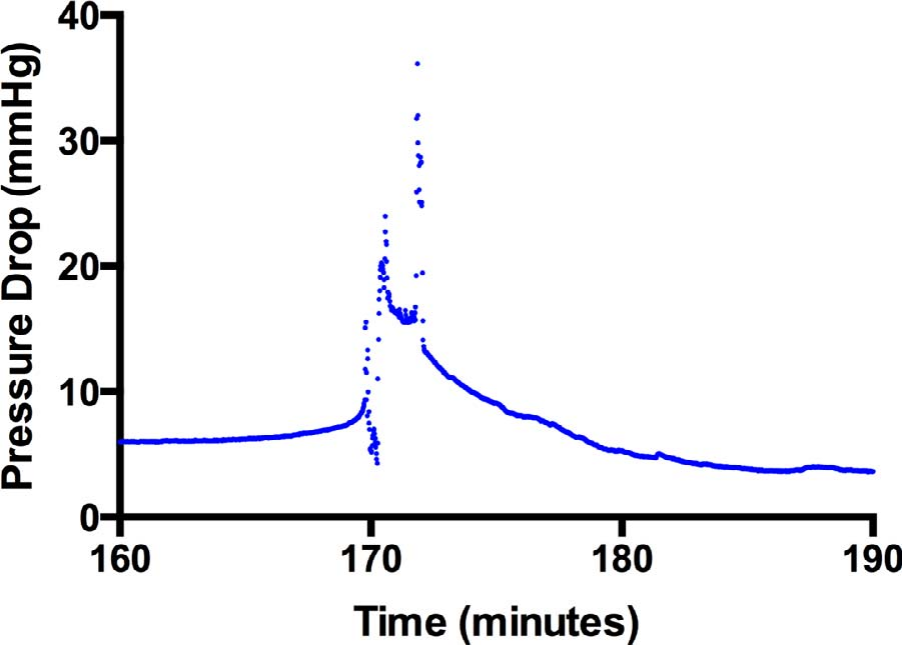
Representative example of a pressure spike when the device was inserted via the ab interno approach. Data were recorded at 1-second intervals, and it is observed that the spike takes approximately 20 minutes to return to baseline whereas any sudden changes to an outflow pathway would resolve very quickly and less gradually, suggesting this phenomenon is related to bleb formation.

The ab interno approach increased outflow resistance by 0.80 ± 0.11 mm Hg/μL/min vs. ab externo 0.42 ± 0.05 mm Hg/μL/min (Fig. 2), and contribution to IOP was 90.4% greater (1.60 ± 0.22 vs. 0.84 ± 0.1 mm Hg; Fig. 3). Statistical analysis demonstrated a significant difference between the two approaches by using an unpaired two-tailed *t*-test (*P* = 0.004). It was observed that although the resulting subconjunctival bleb following the ab externo approach tended to match the area of dissection created using Westcott scissors, the bleb was smaller and more variable following the ab interno approach. Once an outflow pathway was established at baseline and following insertion of the PEEK tubing via the ab interno or ab externo approach, there was minimal variation in data recording for each individual eye (Fig. 3). However, it was also observed that in several ab interno eyes, the IOP peaked to levels greater than 21 mm Hg on several occasions, with each peak getting smaller during the course of the experiment (Fig. 4).

## Discussion

The development of MIGS has stemmed from the need for devices that are easier to insert and are associated with less postoperative complications than conventional GDDs to reduce patient morbidity. MIGS devices that drain into the subconjunctival space need some form of flow control mechanism because otherwise there is a risk of hypotony. The Xen gel stent and InnFocus microshunt control aqueous humor outflow based on the Hagen-Poiseuille equation, where the outflow resistance is a result of the lumen diameter and tube length.^9^ However, whereas the Xen gel stent is designed to provide 6 to 8 mm Hg of resistance at an aqueous flow rate of 1.2 μL/min, reported outcomes at 1 year are far greater at levels closer to 15 mm Hg, which would suggest that there are factors other than just the device lumen dimensions that affect clinical outcomes.^12^,^13^ The IOP levels obtained following Xen gel stent insertion are also greater than those obtained with the InnFocus microshunt.^11^

One of the most significant differences between the two devices is the method of insertion. MIGS was a term that was initially coined for devices that were inserted via an ab interno approach but have since also included devices inserted via an ab externo approach. Each approach has its advantages and disadvantages. The ab interno approach allows for the insertion of the MIGS device at the time of phacoemulsification surgery, therefore reducing tissue manipulation or the need for sutures. Although preoperative mitomycin C (MMC) can be injected before implantation, it does not have the same control as if it were used in an ab externo approach as per the Moorfields Safer Surgery System.^14^ Therefore, there is a significant risk that the Xen gel stent may not achieve adequate long-term outcomes following implantation due to inadequate optimization of the wound healing response.

One of the advantages of the ab externo approach is that it requires less manipulation within the anterior chamber than the ab interno approach. The ab externo approach, although requiring conjunctival dissection, allows the surgeon to direct aqueous humor outflow posteriorly in a more predictable fashion than the ab interno approach. Subconjunctival blebs are, therefore, more likely to be diffuse with a lower risk of long-term failure.

We observed during the study that the resulting blebs following the ab interno approach were smaller and more variable. This may, therefore, play a greater role in controlling IOP, and it was observed that the difference in pressure drop across eyes where the tube was inserted via an ab interno approach was greater and more variable between eyes than via an ab externo approach (Fig. 3). We also noted that in some cases, IOP “spiked” to levels greater than 21 mm Hg (Fig. 4), whereas this was not observed in any of the cases via the ab externo approach. It was believed that the spikes were due to a valve-like mechanism at the posterior border of the subconjunctival bleb created via the ab interno approach. As fluid drained posteriorly past the bleb, the maximum level of the spikes would decrease and be further apart as bleb formation became more stable and more tissue dissected posteriorly by the preceding spikes. Any sudden changes to the outflow pathway would be observed but would return to baseline. Indeed, changes to the bleb dimensions will result in changes to the bleb volume and it would take significantly longer for outflow resistance to respond to these changes. In one eye (Fig. 4), it took approximately 20 minutes for the pressure drop to go back to its original value due to the elastic nature of the conjunctiva. Although these spikes are important from an IOP control perspective, they also provide evidence that pursing of the subconjunctival bleb in the early postoperative period may play a role in helping to monitor IOP. We have also performed studies and developed theoretical models that explain how the appearance of subconjunctival blebs in the early postoperative period may be used to develop clinical grading systems to help improve surgical outcomes following surgery (Bouremel Y, et al. *IOVS*. 2017;58:ARVO E-Abstract 5576).^15^ These pressure spikes did not occur in all eyes, and further studies are currently underway to shed more light on why these results were observed.

Limitations of the study include that the experiments were performed using ex vivo rabbit eyes. The size of the eye and the tissue will not be the same as a human subject intraoperatively and, therefore, it is difficult to establish for certain the implications of the results obtained. The microfluidic setup and method of implantation are different to that of the current MIGS device and may affect flow parameters, although it should be possible to compare devices based on a cylindrical lumen design given that outflow resistance between devices is governed by lumen length and diameter, respectively. We also did not dissect the conjunctival plane with fluid to simulate the preoperative injection of MMC during the ab interno approach. Although an MMC injection may play a role in altering the tissue plane and, therefore, the effect of the bleb on IOP control compared to if an injection was not used, injections are inherently more random and less predictable than a standardized approach to conjunctival dissection as per an ab externo approach. It is, therefore, likely our observations are similar to those we might expect in vivo, and we believe one of the key findings of our results is the unpredictability of the bleb development with devices implanted via the ab interno approach with preimplantation subconjunctival injection of MMC.

Although it is likely that the surgical approach is not the only reason why we observed a difference in surgical outcomes, we did find a statistically significant difference in outflow resistance between the two approaches. Further studies are necessary to assess its significance in an in vivo environment and to assess the nature of bleb formation between the two different techniques and IOP control. We believe that these results have implications for long-term postoperative outcomes and could be an important consideration for the development of future MIGS devices.
